# Double‐blind, randomised, placebo‐controlled trial on the effect of L‐carnitine and L‐acetylcarnitine on sperm parameters in men with idiopathic oligoasthenozoospermia

**DOI:** 10.1111/and.13267

**Published:** 2019-03-15

**Authors:** Sava Micic, Natasa Lalic, Dejan Djordjevic, Nebojsa Bojanic, Natasa Bogavac‐Stanojevic, Gian Maria Busetto, Ashraf Virmani, Ashok Agarwal

**Affiliations:** ^1^ Andrology Department Uromedica Polyclinic Belgrade Serbia; ^2^ Urologic Clinic Clinical Center of Serbia Belgrade Serbia; ^3^ Department of Medical Biochemistry Faculty of Pharmacy University of Belgrade; ^4^ Gynecology and Urology La Sapienza University Rome Italy; ^5^ Innovation‐Research and Development Sigma tau Health Science Utrecht The Netherlands; ^6^ American Center for Reproductive Medicine Andrology Center Cleveland Ohio

**Keywords:** L‐acetylcarnitine, L‐carnitine, male infertility, oligoasthenozoospermia, sperm parameters

## Abstract

Carnitine is essential for energy metabolism and spermatozoa maturation. Combining L‐carnitine and L‐acetylcarnitine with micronutrients has been investigated as a treatment for infertility in men. We evaluated the effects of a therapeutic formulation, Proxeed Plus, on sperm parameters in oligoasthenozoospermic men. This prospective, randomised, double‐blind, placebo‐controlled clinical trial involved 175 males (19–44 years) with idiopathic oligoasthenozoospermia who failed to impregnate their partners (12 months). Males received Proxeed Plus or placebo for 3 and 6 months. Sperm volume, progressive motility and vitality significantly (*p* < 0.001) improved after 6 months compared to baseline. Sperm DNA fragmentation index significantly decreased compared to baseline (*p* < 0.001) and the 3‐month therapy (*p* = 0.014) in treated men. Increased seminal carnitine and α‐glucosidase concentration also positively correlated with improved progressive motility. Decreased DNA fragmentation index was the good predictor of progressive sperm motility >10%, and simultaneous measurement of changes in sperm vitality and DNA fragmentation index gave the highest probability of sperm motility 10% (AUC = 0.924; 95% CI = 0.852–0.996; *p* < 0.001). Logistic regression analyses revealed DNA fragmentation index decrease as the only independent predictor of sperm motility 10% (OR = 1.106; *p* = 0.034). We have demonstrated the beneficial effects of carnitine derivatives on progressive motility, vitality and sperm DNA fragmentation. Combining metabolic and micronutritive factors is beneficial for male infertility.

## 1 INTRODUCTION

Male fertility issues are major contributors to infertility in couples, accounting for approximately 50% of these cases (Agarwal, Mulgund, Hamada, & Chyatte, 2015). The causes of male infertility remain unknown, and are ambiguous in almost 40%–60% of these patients (Gabrielsen & Tanrikut, 2016). Due to the unclear causes of idiopathic male infertility, the main challenge involves the selection of a therapeutic modality. Since patients with oligoasthenozoospermia represent a very diverse group, treating this particular group is not an easy task. As a possible treatment option, some food supplements may improve seminal fluid conditions, provide energy to male germ cells and protect these cells from oxidative stress. Since spermatogenesis is an energy‐intensive process, it requires a highly balanced supply of minerals, antioxidants and nutrients.

Oxidative stress, sperm apoptosis and DNA damage significantly contribute to the pathogenesis of male infertility (Agarwal & Said 2004). Sperm DNA damage is associated with reduced fertility and increased frequency of spontaneous abortions, and affects embryo quality (Lewis et al., 2013). Moreover, it is considered a potential predictor of fertility, and its detection has repeatedly been described as a prognostic parameter of male fertility in various assisted reproduction techniques and andrological diagnostics (Agarwal, Majzoub, & Esteves, 2016; Bungum et al., 2007; Cho, Agarwal, Majzoub, & Esteves, 2017; Henkel et al., 2004).

L‐carnitine (LC) plays a major role in the oxidation of long‐chain fatty acid, and its active form, L‐acetylcarnitine (ALC), protects mitochondria from metabolic toxins via its antioxidant effects. ALC also stabilises cell membranes and exerts anti‐apoptotic actions (Abdelrazik, Sharma, Mahfouz, & Agarwal, 2009; Kerner & Hoppel, 1998; Vanella et al., 2000; Zhou, Liu, & Zhai, 2007). LC absorbed by epididymal cells is released into the epididymal lumen and luminal part of the seminiferous epithelium. The concentration of LC in the epididymal lumen and in sperm cells is approximately 2,000‐fold greater than in the circulating serum, implying it plays a very important role in sperm maturation, metabolism and motility (Mongioi et al., 2016).

Since the epididymis plays an essential role in sperm maturation, progressive motility and the achievement of fertilisation capacity by male germ cells, several markers can be used to evaluate epididymal function, including LC (Cooper, 1990; Haidl & Schill, 1997) or α‐glucosidase (Cooper, Yeung, Nathan, & Nieschlag, 1988; Garcia Diez, Esteban Ruiz, & Vilar, 1992).

Positive correlations between seminal carnitine concentration, sperm count and motility have been demonstrated in clinical studies (Matalliotakis et al., 2000; Menchini‐Fabris, Canale, Izzo, Olivieri, & Cartelloni, 1984; Mongioi et al., 2016). Several randomised studies assessing the effects of different daily doses of carnitine on sperm parameters in idiopathic oligoasthenozoospermic men have been published. In two studies, which included 100 and 60 infertile men, respectively (Lenzi et al., ) sperm concentration, motility and linearity were shown to increase. Similar results were obtained by Balercia et al. (2005) who examined 60 men with oligoasthenozoospermia. In 52 men treated with clomifene, carnitine resulted in higher sperm concentration and motility (Moradi, Moradi, Alemi, & Ahmadnia, 2010). In an open study comprising of 114 oligoasthenozoospermic men, Busseto et al. (2012) observed increased sperm motility, while Sigman, Glass, Campagnone, & Pryor (2006) found no effects on sperm motility or total motile count in a group of 21 asthenozoospermic men.

In light of these positive indications, we aimed to evaluate the effect of a combined formulation, Proxeed Plus, on sperm and seminal biochemical parameters, in a randomised, double‐blind, placebo‐controlled clinical trial with idiopathic oligoasthenozoospermic men who failed to impregnate their wives over the past 12 months.

## 2 PATIENTS AND METHODS

### 2.1 Patients and study design

This prospective, randomised, double‐blind, placebo‐controlled clinical trial was approved by the institutional review board of the Association of Serbian Private Healthcare Providers, Belgrade, Serbia (Institute Ethical Approval Number PXP‐001B/24). After obtaining written informed consent from participants, the study was conducted with 175 infertile men with idiopathic oligoasthenozoospermia in the Department of Andrology‐Uromedica Polyclinic, Association of Serbian Private Healthcare Providers, Belgrade, Serbia. Patients (mean age, 31.5 years; 19–44 years) were enrolled between December 2014 and January 2016.

In this study, the effect of the test formulation, Proxeed Plus, was investigated in idiopathic oligoasthenozoospermic men who were having trouble impregnating their partners for at least 12 months.

The applied test formulation consisted of 1,000 g LC, 0.5 g ALC, 0.725 g fumarate, l g fructose, 50 mg citric acid, 10 mg zinc, 20 mg coenzyme Q10, 50 µg selenium, 90 mg vitamin C, 200 µg folic acid and 1.5 µg vitamin B12. The placebo was made with the excipients (sucrose, silica [anti‐caking], lemon flavour, acesulfame K [E950, sweetener]) of the supplementation without the active substances.

After 2 months of “wash‐out” (period without any therapy), treatment occurred for 6 months with Proxeed Plus (125 patients) or placebo (50 patients), followed by control semen analyses 3 and 6 months after therapy initiation (T3, T6). Patients received either test formulation, Proxeed Plus, or placebo (two times per day) in a randomised, double‐blind fashion.

All participants met the inclusion criteria of one semen analysis that demonstrated either total sperm number ≤15 million per ml; progressive motility <32%; normal viscosity and normal leucocytes number (<1 × 10^6^/ml); total ejaculate volume 1.0 ml; sperm vitality ≤58% live; normal sperm morphology <4% (according to WHO, 2010).

The following female partners were included in the study:

- fertile partners with regular menstrual cycles, and younger than 40 years;
- infertile partners provided no fertility‐ related procedures such as artificial insemination (AI), in vitro fertilisation (IVF) or intracytoplasmic sperm injection (ICSI) were planned for the next 90 days.

Exclusion criteria for the male participants were as follows: motility <5%; sperm concentration <1 × 10^6^/ml; history of undescended testes; subjects with known hypersensitivity to ingredients in Proxeed Plus; endocrine disorders affecting the hypothalamic–pituitary axis; history of post‐pubertal mumps; presence of anti‐sperm antibodies; history of endocrine disease; autoimmune disease, cystic fibrosis, or testicular cancer; leucocytospermia, leucocyte count >1 × 10^6^/ml; use of antioxidant agents or vitamins within the 8 weeks prior to inclusion in the study (for subjects using vitamin supplementation, an 8‐week wash‐out period was required prior to inclusion in the study); use of vitamin or natural treatment for infertility at any time; history of taking any therapy for infertility within the last 2 months including over‐the‐counter treatment and vitamin supplementation; history of excessive consumption of alcohol 90 days prior to the start of the trial; subjects involved in other clinical trials.

### 2.2 Preparation and analyses of semen samples

Semen samples were obtained after 3 days of sexual abstinence at the Andrology Department of Uromedica Polyclinic, Belgrade, Serbia. Samples were liquefied at 37°C and then analysed according to the World Health Organization (WHO) criteria (WHO, 2010). Remnants of liquefied semen samples were immediately centrifuged at 1,000 *g* for 10 min. The seminal plasma was divided into several aliquots and frozen at −80°C for biochemical analysis.

Samples were scored to evaluate motility; the results were expressed as motility and progressive motility. Sperm DNA defragmentation index (DFI) was determined by the sperm chromatin dispersion test, Halosperm (Halotech DNA, S.L. Madrid, Spain). To determine α‐glucosidase activity, a 20 µl aliquot of seminal plasma was incubated for 2 hr with p‐nitrophenyl‐α‐D‐glucopyranoside at 37°C (pH 6.8); this is because α‐glucosidase specifically hydrolyses p‐nitrophenyl‐α‐D‐glucopyranoside into p‐nitrophenol. The reaction was stopped by adding 1 ml 0.02 M NaOH. The quantity of p‐nitrophenol was measured using a biochemistry analyzer (ContecBc 300, Beijing, China) at a wavelength of 405 nm.

Seminal LC concentration was determined enzymatically using a UV test (Roche Diagnostics GmbH, Penzberg, Germany).

### 2.3 Statistical analysis

Normal distribution of the data was tested using Shapiro–Wilk test. Since distribution of the data was not normal, nonparametric tests were employed. Continuous variables are presented as median values and interquartile ranges while categoric data are presented as absolute or relative frequencies. Variables were compared using Friedman test and post hoc Wilcoxon Signed Rank test, with Bonferroni corrections, or in case of monitoring of changes in two points, only using Wilcoxon Signed Rank test. Differences between categoric variables were analysed using McNemar–Bowker test if the change was monitored in three points (for *k* × *k* contingency table) or McNemar test if the change was monitored in two points (for 2 × 2 contingency tables).

We calculated per cent of examined parameters change between T0 and T6. For parameters which levels decreased during the therapy (e.g., DFI values), we applied formula ([T0 − T6]/T0 × 100), and for parameters which level increases in second point (e.g., sperm vitality, seminal carnitine concentration, α‐glucosidase activity), percentage changes are calculated by ([T6 − T0]/T6 × 100) formula. Per cent of changes calculated for examined parameter was subject for statistical analyses. Correlation between determined parameters was established using Spearman's rank correlation. Binary logistic regression analysis was used for testing of probability for increased motility of spermatozoa by 10%, 20% or 30%. We used receiver operating characteristic curve (ROC) analysis to evaluate biomarker use in specific clinical decisions. According to Swets (1988), criteria for areas under ROC curves (AUC), diagnostic tests were categorised in the group with poor accuracy 0.5–0.7; satisfactory accuracy for certain purposes 0.7–0.8; good accuracy 0.8–0.9 and excellent accuracy >0.9. Variables with best predictive properties were combined in order to produce a model with the best discriminatory properties and areas under curves were presented as C‐statistic.

A *p*‐value ≤0.05 was considered statistically significant. Statistical data processing was performed using Statistical Package for the Social Sciences PASW^®^ Statistic v. 22 (Chicago, IL, USA) software.

## 3 RESULTS

Sperm parameters were tested before (T0), as well as three (T3) and six (T6) months after the start of Proxeed Plus or placebo therapy. The results obtained are presented in Table 1A,B.

**Table 1:** Sperm parameters before therapy and after 3 and 6 months of (A) Proxeed Plus therapy. (B) Placebo treatment

(A) Sperm parameters	T0	T3	T6	*p*
Volume, ml	3.00 (2.13–4.70)	3.20 (2.50–4.40)*	3.60 (2.60–4.50)*	0.020
DFI, %	38.50 (32.00–48.70)	35.50 (25.50–44.00)*	31.00 (25,00–41,00)[Link], [Link]	<0.001
Progressive sperm motility, %	28.0 (12.0–38.0)	30.0 (12.0–39.0)	31.0 (20.0–41.0)**	<0.001
Vitality, %	0.52 (0.43–0.60)	0.57 (0.46–0.64)	0.56 (0.47–0.65)*	0.002
Seminal glucosidase U/L	25.40 (20.00–42.88)	N.D.	32.50 (23.00–42.83)	0.002
Carnitine, µmol/L	724.6 (626.45–800.48)	N.D.	782.8 (686.55–926.25)	<0.001

(B) Sperm parameters	T0	T3	T6	*p*
Volume, mL	3.3 (2.9–3.9)	3.1 (2.85–3.70)	3.40 (3.00–3.90)	0.127
DFI, %	37.00 (34.50–41.00)	38.00 (35.50–40.50)	38.00 (34.00–39.00)	0.306
Progressive sperm motility, %	23.0 (18.0–28.0)	24.0 (19.5–29.0)	24.00 (20.0–28.0)	0.098
Seminal glucosidase U/L	23.50 (20.90–27.95)	N.D.	23.50 (21.6–27.95)	0.084
Carnitine, µmol/L	710.0 (682.45–733.40)	N.D.	709.5 (699.7–722.6)	0.580

Notes

N.D.: not determined.

Data are presented as median values (25th–75th percentiles). Comparison was performed by Friedman test (for three dependent populations).

*Significant difference compared to the values obtained before therapy.

**Significant difference compared to the values obtained 3 months after therapy; Post hoc test is the Wilcoxon Signed Ranks test with Bonferroni correction.

Statistically significant differences were observed for ejaculate volume, progressive motility, vitality and DFI between the three different time points. There were also significant differences in seminal carnitine concentration and seminal α‐glucosidase activity before therapy (T0), and after 6 months of therapy (T6).

Values of most sperm parameters such as ejaculate volume (*p* = 0.001), progressive sperm motility (*p* < 0.001) and sperm vitality (*p* = 0.002) were significantly higher at T6 compared to T0. A significant increase in progressive motility (*p* = 0.004) was only observed after 6 months of therapy (T6). DFI significantly decreased from T0 to T3 (*p* < 0.001) with an additional decrease after 6 months of treatment (T6: *p* = 0.01). In contrast, subjects who received the placebo showed no changes after 3 and 6 months of treatment (Table 1B).

We calculated median changes for DFI, seminal carnitine, vitality and α‐glycosidase activity between T0 and T6. Median decrease in DFI levels was 21%, and median increase in seminal carnitine, vitality and α‐glucosidase activity was 6.5%, 6% and 8.3% respectively.

We examined the per cent of patients with three levels of progressive sperm motility at the three points (T0, T3, T6), and present sperm motility as a percentage (Table 2). The proportion of men with progressive sperm motility 10, between 10% and 20%, and 20% at the three points (T0, T3, T6) displayed significant changes (*p* = 0.001).

**Table 2:** Percentage of patients with different levels of progressive sperm motility before and during Proxeed Plus therapy

Sperm motility	T0	T3	T6	*p*
<10%	20.9	16.7	16.6	0.001
10%–20%	17.6	16.7	10.0	
>20%	61.5	66.7	73.4	

Note

Progressive sperm motility is presented as a percentage. Comparison was performed by McNemar–Bowker test.

In addition, we examined the change in the percentage of subjects with progressive sperm motility <10% and >10% between the time points, T0 and T6 (2 × 2 contingency tables). The percentage of subjects with sperm motility >10% was not significantly higher at T6 (83.4%) when compared to T0 (79.4%; *p* = 0.267). A similar analysis was performed to test the changes in percentage of men with progressive sperm motility above and below 20% and percentage of men with sperm motility above and below 30%. A statistically significant increase in the percentage of males with progressive sperm motility above 20% was observed at T6 (73.4%) compared to T0 (61.55%; *p* = 0.007). After investigating the proportion of men with progressive sperm motility higher than 30%, an increase was not observed at T6 (51.1%) compared to T0 (44.4%; *p* = 0.286).

We analysed the correlation between progressive sperm motility and percentage change between T0 and T6. The higher reduction of DFI between the time points (T0 versus T6) was associated with greater sperm motility (*R* = 0.269, *p* = 0.024). This finding is similar to seminal carnitine concentration and sperm vitality. A higher increase in seminal carnitine and vitality resulted in a greater progressive sperm motility (*R* = 0.274, *p* = 0.023 for carnitine; *R* = 0.033, *p* = 0.008 for sperm vitality). Changes in other parameters between the two periods (T0 versus T6) had no significant influence on sperm motility.

Using logistic regression analysis, we calculated the probability of a change in the tested parameters between T0 and T6 to predict the likelihood of males having a sperm motility 10% at T6 (Table 3; i.e., predictive capability of change in the studied parameters between the two periods was evaluated to identify men with sperm motility 10% at T6). A 1% increase in sperm vitality increased the probability of a 10% sperm motility by 1.064‐fold after 6 months of therapy. In contrast, a 1% decrease in DFI resulted in a 1.105‐fold increase in the probability of a higher than 10% sperm motility. Positive changes in α‐glucosidase and carnitine concentration were associated with the increased likelihood of a 10% sperm motility; however, their influence is marginally significant (*p* = 0.048 for α‐glucosidase and *p* = 0.046 for carnitine).

**Table 3:** Influence of per cent changes in the studied parameters on a greater than 10% progressive increase in sperm motility after 6 months of therapy

Sperm parameters	OR (95% CI)	AUC (95% IP)
Carnitine increase between T0/T6 (%)	1.041 (1.001–1.082) *p* = 0.046	0.713 (0.591–0.815) *p* = 0.009
α‐Glucosidase activity increase between T0/T6 (%)	1α0.021 (1.001–1.042) *p* = 0.048	0.688 (0.571–0.789) *p* = 0.020
Volume increase between T0/T6 (%)	1.000 (0.991–1.008) *p* = 0.911	0.604 (0.481–0.718) *p* = 0.256
Sperm vitality increase between T0/T6 (%)	1.064 (1.016–1.115) *p* = 0.009	0.836 (0.728–0.913) *p* < 0.001
DFI decrease between T0/T6 (%)	1.105 (1.035–1.178) *p* = 0.003	0.793 (0.686–0.877) *p* < 0.001

Notes

AUC: area under ROC curve; CI: confidence interval; OR: odds ratio.

Percentage change between T0 and T6.

Receiver operating characteristic curve analysis was used to test the diagnostic capacity of a percentage change in the studied parameters to identify individuals with 10% sperm motility. Changes in sperm vitality showed the best diagnostic characteristics as AUC was 0.8 (based on Sweats criteria, this value defines good diagnostic capacity). Percentage decrease in DFI is less accurate compared to spermatozoa vitality, but its diagnostic capacity may also be defined as good. Carnitine increase showed a satisfactory capacity to detect individuals with 10% sperm motility (its AUC is moderately 0.7). The AUC for increase in α‐glucosidase activity was not satisfactory, and the increase in the diagnostic capacity of volume was not statistically significant.

The parameter changes between T0 and T6 that are related to 20% (Table 4) and 30% (Table 5) progressive motility were analysed in a similar manner. Increased sperm vitality showed the best predictive values. Males with a parameter increase of 1% had a 1.119‐fold higher probability of a spermatozoa progressive motility 20% after 6 months of therapy when compared to individuals lacking a record of increase in this parameter. Additionally, the diagnostic capacity of this parameter is included in the list of tests possessing excellent characteristics (AUC 0.9). Per cent DFI decrease is the only other parameter that may predict a sperm motility 20% (OR = 1.064); however, its capacity to diagnose individuals with a sperm motility 20% is not relevant from a clinical viewpoint (AUC 0.7).

**Table 4:** Influence of percentage change in studied parameters after 6‐month therapy on progressive increase in sperm motility greater than 20%

Sperm parameter T0/T6 (%)	OR (95% CI)	AUC (95% CI)
Carnitine increase between T0/T6 (%)	1.030 (1.000–1.062) *p* = 0.052	0.636 (0.500–0.772) *p* = 0.083
α‐Glucosidase activity increase between T0/T6 (%)	1.064 (1.014–1.117) *p* = 0.011	0.689 (0.542–0.836) *p* = 0.014
Volume increase between T0/T6 (%)	0.995 (0.989–1.002) *p* = 0.154	0.500 (0.344–0.656) *p* = 0.995
Sperm vitality increase between T0/T6 (%)	1.119 (1.052–1.190) *p* < 0.001	0.908 (0.831–0.984) *p* < 0.001
DFI decrease between T0/T6 (%)	1.015 (1.000–1.030) *p* = 0.054	0.671 (0.526–0.817) *p* = 0.029

Notes

AUC: area under ROC curve; CI: confidence interval; OR: odds ratio.

Percentage change between T0 and T6

**Table 5:** Influence of per cent changes in the tested parameters on a greater than 30% progressive increase in sperm motility after 6 months of therapy

Sperm parameter	OR (95% CI)	AUC (95% CI)
Carnitine increase between T0/T6 (%)	1.024 (1.001–1.049) *p* = 0.049	0.617 (0.482–0.752) *p* = 0.096
α‐Glucosidase activity increase between T0/T6 (%)	1.051 (1.006–1.097) *p* = 0.026	0.541 (0.408–0.673) *p* = 0.557
Volume increase between T0/T6 (%)	0.998 (0.992–1.005) *p* = 0.633	0.650 (0.538–0.762) *p* = 0.080
Sperm vitality increase between T0/T6 (%)	1.008 (0.992–1.025) *p* = 0.329	0.525 (0.391–0.660) *p* = 0.722
DFI decrease between T0/T6 (%)	1.012 (1.001–1.024) *p* = 0.042	0.556 (0.422–0.690) *p* = 0.556

Notes

AUC: area under ROC curve; CI: confidence interval; OR: odds ratio.

Percentage change between T0 and T6.

Significant predictors of a 30% progressive motility were the increase in seminal carnitine concentration and α‐glucosidase activity, and the decrease in DFI (Table 6). None of the parameters tested showed satisfactory diagnostic characteristics in the detection of sperm motility 30%.

**Table 6:** Diagnostic characteristics of changes in the parameter values tested to identify individuals with sperm motility greater than 10% or 20%

Sperm parameter	Cut‐off for motility	Se	Sp	PPV	NPV
Carnitine increase between T0/T6 (%)^a^	7.7%	53.3	90.0	97.2	22.5
a‐Glucosidase increase between T0/T6 (%)^a^	3.0%	72.7	81.8	96.0	33.3
Volume increase between T0/T6 (%)^a^	5.9%	55.0	100	100	20.0
Sperm vitality increase between T0/T6 (%)^b^	5.9	61.4	100	100	37.1

Notes

Se: sensitivity; Sp: specificity; PPV: positive predictive value; NPV: negative predictive value.

a Detection of individuals with sperm motility greater than 10%.

b Detection of individuals with sperm motility greater than 20%.

For each parameter change demonstrating good diagnostic accuracy (AUC 0.7), we determined their optimal cut‐off values to discriminate subjects with sperm motility 10% from those with 10% sperm motility. A similar analysis was performed to discriminate patients with sperm motility 20% from those with 10% motility (Table 6).

For sperm vitality per cent increase after 6 months, the optimal cut‐off value for men with 10% sperm motility compared to those with a value 10% was 5.9% (PPV = 100%). The same results were obtained when men with sperm motility 20% were discriminated from those with 20% motility. For DFI changes, the optimal cut‐off value when subjects with sperm motility 10% were discriminated from those with 10% sperm motility was 3% (PPV = 96%). If DFI decreased by 3% after 6 months of therapy, there was a 96% chance that the patient would have a sperm motility 10%. For carnitine increase after 6 months, the optimal cut‐off value to detect men with a sperm motility 10% was 7.7%. Among those men who had an increased carnitine per cent 7.7% after 6 months of therapy, probability of sperm motility 10% was 97.2% (PPV = 97.2%).

In addition, we combined the parameters to better identify individuals with sperm motility 10% or 20%. Simultaneous changes in sperm vitality and DFI after 6 months of therapy were demonstrated to reveal the highest diagnostic accuracy when used to detect individuals with a sperm motility 10%; diagnostic accuracy: AUC = 0.924, 95% CI (0.852–0.996), *p* < 0.001. Application of the same analysis to identify individuals with a sperm motility 20% did not reveal an increase in accuracy after combination compared to determining individual changes in sperm vitality.

## 4 DISCUSSION

Increasing male fertility problems, which are demonstrated by decreases in sperm concentration and the motility of spermatozoa, have been reported in Western countries (Levine et al., 2017) and India (Adiga, Jayaraman, Kalthur, Upadhya, & Kumar, 2008).

Recent studies have indicated the important role of nutrients, vitamins and minerals in sperm health (Balercia et al., 2005; Lenzi et al., 2003). LC and ALC are present in high concentration in the epididymis, and play crucial roles in the development, maturation and metabolism of spermatozoa (Radigue, Es‐slami, & Soufir, 1996) by improving sperm motility, and displaying antioxidant and anti‐apoptotic properties for the stabilisation of the spermatozoa cell membrane (Cheah & Yang, 2011).

In this study, we aimed to investigate the effects of combination therapy using metabolic substances (LC and ALC), antioxidants and vitamins in a randomised, double‐blind, placebo‐controlled clinical trial comprising of 175 infertile men with idiopathic oligoasthenozoospermia. We found a significant increase in seminal carnitine concentration after 6 months of therapy, and a significant positive association between carnitine and progressive spermatozoa sperm motility. Interestingly, higher progressive motility was found in the group of patients with higher basal carnitine concentration (before therapy). These results align with the report of lower seminal carnitine levels in infertile men than in fertile controls (Gurbuz, Yalti, FiCicoglu, & Zehir, 2003; Matalliotakis et al., 2000). Our data further demonstrated that an increase of 7.7% in seminal carnitine levels from basal value is a good predictor of sperm motility recovery. This cut‐off value was also significantly associated with the increase in progressive sperm motility. These findings also align with that found in other studies (DeRosa et al., 2005; Lenzi et al., 2003). In a placebo‐controlled, double‐blind randomised study where infertile men were treated with 2 g/day LC and 1 g/day ALC for 6 months, Lenzi et al. (2004) observed significant improvements in sperm motility. Similarly, a study including 30 normozoospermic and 30 idiopathic asthenozoospermia men treated with 2 g/day LC for 3 months showed sperm motility recovery in the group with normal values for phospholipid hydroperoxide glutathione peroxidase, a marker of mitochondrial structure and function (Garolla et al., 2005).

A study with 60 idiopathic oligoasthenozoospermia men treated for 6 months with either 3 g LC and 3 g ALC, or 2 g LC and 1 g ALC, or placebo (Balercia et al., 2005), showed clear beneficial effects on sperm kinetics; however, a difference was not found relative to progressive sperm motility. Lombardo et al. (2010) reported improved total sperm motility and forward motility after 3 months of therapy with Proxeed NF (LC 250 mg/day and ALC 75 mg/day) in a group of 32 infertile men (sperm concentration, 10–60 mill/ml; total motility, 5%–40%; forward motility, <15%). Two studies (Ahmed, Ahsan, Iqbal, & Burney, 2017; Matalliotakis et al., 2000) found a significant positive correlation between seminal carnitine levels and sperm motility.

In contrast, Sigman et al. (2006), in a double‐blind, placebo‐controlled study involving a small number of patients treated with 2 g LC and 1 g ALC daily for 24 months, reported no significant improvement in sperm motility. Recently, Busetto et al. (2018) analysed 104 oligo‐ and/or astheno‐ and/or teratozoospermic men, with and without varicocele, who received Proxeed Plus or placebo for 6 months based on a randomisation schedule. A significant increase in total sperm count and progressive sperm motility was demonstrated in the supplemented group, with and without varicocele; no difference was observed in progressive sperm motility after 6 months of treatment between men with and without varicocele. They concluded that oxidative stress has a negative effect on semen parameters, and using carnitine and other substances can serve as an efficacious therapy to improve male fertility.

α‐Glucosidase, which is normally present in semen together with LC and glycerophosphocholine, is a sensitive marker of epididymal function (Cooper, Yeung, Nashan, Jockenhövel, & Nieschlag, 1990). We found a significant increase in α‐glucosidase concentration after 6 months of therapy, which aligns with the higher levels of α‐glucosidase observed in fertile compared to infertile men by Zopfgen et al. (2000), and the positive correlation between α‐glucosidase activity and motility by Roaiah et al. (2007). Some studies did not observe a difference in α‐glucosidase activity relative to spermatozoa motility (Krause & Bohring, 1999; Lenzi et al., 2003; Said et al., 2009).

Standard methods for ejaculate analyses are often insufficient to predict successful pregnancy; yet, increased sperm DNA fragmentation has been demonstrated as a good and reliable predictor of male infertility as well as fertilisation and pregnancy (Bungum et al., 2007; Simon et al., 2013; Zhao et al., 2014). Studies in the last decade have indicated a high per cent of DNA fragmentation in the general population and during IVF procedures (Alshahhrani et al., 2014; Cho et al., 2017; Henkel et al., 2004; Zhao et al., 2014). In our study, we noticed a significant decrease in DFI after 3 months of therapy, followed by a further significant decrease after an additional 3 months of therapy (T6). We also found that if DFI decreased by 1%, the probability of a sperm motility higher than 10% increased 1.105‐fold.

We employed ROC analyses to determine the optimal seminal parameters and sperm motility 10% or 20%. Results showed that a 3% decrease in DFI after 6 months of therapy predicted a progressive motility 10% with a probability of 96%. In addition, we demonstrated that the only independent predictor of a progressive motility 10% and 20% for spermatozoa is a decrease in DFI. However, the capacity of DFI values in the diagnosis of motility 20% is not relevant from a clinical viewpoint. Our data merely imply that a change in DFI is moderately associated with the increase in progressive motility after 6 months of Proxeed Plus therapy.

Scarce data exist regarding the effects of anti‐oxidative therapy in infertile men, and DFI values. Varicocelectomy in infertile men led to a significant decrease in DFI values (Smit et al., 2013). Analysis of 38 couples undergoing IVF revealed that the male partners had increased DFI%, but after two months of therapy with vitamin C and vitamin E, a DFI% decrease in 76% of males and an increased number of successful pregnancies were observed (Greco et al., 2005). In a recent Cochrane analysis, a positive effect owing to oral anti‐oxidative therapy and successful pregnancies were suggested; in this analysis, only two papers had investigated DNA fragmentation and the authors reported a 13% decrease in DFI in patients who received anti‐oxidative therapy (Showell et al., 2014).

In this study, we showed a high probability of males achieving progressive motility 10% after 6 months of therapy; the cut‐off value of 5.9% representing the increase in sperm vitality after 6 months of therapy is associated with a progressive motility 10% or 20%. This occurrence is associated with a 100% probability, indicating that sperm vitality is a good predictor of progressive motility. Aside from this, we emphasise that simultaneously determining DFI and vitality has the highest diagnostic validity when used to identify patients with 10% progressive motility of spermatozoa. According to WHO (2010), determining sperm vitality is one of the main parameters in standard semen analysis, and is particularly important in samples containing a high number of immotile spermatozoa.

In this study, after 6 months of therapy with the formulation of LC and ALC, we observed significant beneficial effects in progressive motility, vitality and DNA fragmentation. These results suggest that seminal levels of LC play an essential role in maintaining male fertility. Moreover, these results support the significance of formulations that combine metabolic and nutritive factors as a therapy to treat male infertility and recover the functional capacity of spermatozoa.
